# Digital Health for Breast Care: Patient Satisfaction and Reducing Disparities through Telemedicine

**DOI:** 10.1155/ijbc/1932655

**Published:** 2025-12-07

**Authors:** Yekta Soleimani Jobaneh, Saba Alvand, Nahid Raei, Fataneh Khalaj, Shahpar Haghighat, Ahmad Kaviani

**Affiliations:** ^1^ T.H. Chan School of Medicine, UMass Chan Medical School, Worcester, Massachusetts, USA, umassmed.edu; ^2^ School of Medicine, Tehran University of Medical Sciences, Tehran, Iran, tums.ac.ir; ^3^ Breast Cancer Research Center, Motamed Cancer Institute, ACECR, Tehran, Iran, acecr.ac.ir; ^4^ Department of Surgical Oncology, University of Montreal, Montreal, Canada, umontreal.ca

**Keywords:** breast health, healthcare disparities, patient satisfaction, telemedicine, virtual consultation

## Abstract

**Background:**

Virtual teleconsultation plays a pivotal role in managing diseases requiring long‐term communication between patients and treatment teams, such as breast diseases. The Ruban Virtual Breast Clinic in Iran offers teleconsultation services focusing on nonurgent chronic complaints through offline messaging. This study aimed to evaluate patient satisfaction with these teleconsultation services.

**Methods:**

A comprehensive questionnaire was designed with three sections: identifying the individual interacting with the clinic and prior teleconsultation use; collecting demographic data and reasons for consultation; and assessing satisfaction using 16 items rated on a Likert scale from 1 (*poor*) to 10 (*excellent*). The study included patients who received at least one consultation by a breast surgeon through the Ruban platform.

**Results:**

Of 583 eligible cases, 367 (62.9%) consented to participate. The average satisfaction score was 91.6 out of 100, indicating a high level of patient satisfaction.

**Conclusions:**

The high satisfaction rates suggest that telehealth services, particularly virtual consultations, are feasible and highly acceptable in meeting patients′ healthcare needs. These findings underscore telehealth′s potential to improve access to care, though further research is required to establish its clinical effectiveness.

## 1. Introduction

Telemedicine involves using telecommunications technology to provide medical care and services from a distance. Established as a practice in the 1920s and initially involving two‐way radio communication—with a notable early example being Norway′s Haukeland Hospital′s remote treatment of seafarers—the subsequent decades saw global adoption of this method, and the 1950s and 1960s marked the introduction of two‐way video communication, famously used by NASA astronauts [1, 2].

In December 2019, the World Health Organization (WHO) announced the emergence of SARS‐CoV‐2, commonly known as COVID‐19. By March 11, 2020, the outbreak was declared a global pandemic [3, 4], leading to widespread lockdowns and stringent measures to limit the virus′s spread [5]. Faced with these unprecedented challenges, healthcare providers and patients increasingly relied on telehealth platforms and teleconsultation services [6].

Since then, telemedicine has facilitated remote consultations between healthcare professionals and patients, significantly impacting disease management and medical follow‐ups [7–10]. Notably, telehealth consultations have played a crucial role in managing chronic conditions, including breast cancer [11], inflammatory bowel disease (IBD) [12], rheumatic and musculoskeletal diseases [13], neurological disorders [14–17], and dermatological conditions [18]. Present‐day teleconsultation methods encompass telephone visits, video calls, and text messaging.

In the context of breast health, patients often require regular consultations and checkups. The frequent follow‐up visits required for breast cancer survivors and periodic examinations for various breast conditions, such as breast cancer, high‐risk breast lesions, and idiopathic granulomatous mastitis (IGM), underscore the importance for healthcare policymakers to ensure accessible follow‐up care [19–21].

Telehealth has transitioned from an emerging solution to a mainstream service, spurred by the COVID‐19 pandemic and social distancing mandates. Its popularity and application continue to grow, driven by its potential to ensure accessible follow‐up care and address healthcare disparities [22, 23]. Telehealth provides a transformative approach to making healthcare more accessible and equitable by allowing remote delivery of medical care and health‐related services. This is particularly beneficial for populations traditionally marginalized in healthcare settings, such as those in rural areas, underprivileged communities, and individuals with mobility challenges, enabling them to access specialist consultations, routine checkups, and mental health services without traveling [24, 25].

However, the success of telehealth in reducing disparities depends on understanding and analyzing patients′ acceptance and satisfaction with these services. A group of physicians with specialties related to breast disease management established the “Ruban Virtual Breast Clinic,” focusing on providing consultations for breast disease. This study aims to assess patient satisfaction with the teleconsultation services offered by our virtual breast clinic, marking the first study to utilize offline messaging as a consultation method for patients with nonurgent chronic breast complaints.

## 2. Methods and Materials

This research was conducted as a descriptive cross‐sectional study in 2022. Enrollment in the study commenced upon patient registration via a website or mobile application (Ruban Virtual Breast Clinic), enabling access to an offline text and image‐based medical consultation. Patients articulated their symptoms through text‐based communication and by uploading files pertinent to their additional investigations. In this virtual clinic setup, when a patient requested to contact a physician, the server promptly notified the physician via their private cell phone or email address. Physicians were expected to respond to these consultations at their earliest convenience, ensuring a response time of no more than 24 h. Consultations were conducted either via voice or text messaging. The physicians had the authority to conclude a case once the patient′s recent complaints were adequately addressed.

Given that Ruban operates as a breast clinic, it integrates various subspecialties, including breast surgical oncology, radiation oncology, medical oncology, breast imaging, pathology, genetics, nuclear medicine, internal medicine, gynecology, and lymphology, to offer comprehensive consultations. To address the limited nature of complaints across subspecialties, our study included patients who had received at least one consultation by a breast surgeon.

The study protocol was approved by the Ethics Committee of the Breast Cancer Research Center (Approval Code: IR.ACECR.IBCRC.REC.1400.029). Verbal informed consent was obtained from all participants. The study adhered to the principles outlined in the Declaration of Helsinki.

### 2.1. Instruments

Data registration was achieved by the following instruments:

1. General information checklist: This section consisted of information regarding the individual interacting with the virtual clinic. It included questions about the patient or its relatives (by asking the relationship between the contact person and the patient), any previous experiences with virtual consultations, and the nature of the consultation (categories included new complaints, follow‐up visits, updates on existing conditions, treatment inquiries, and requests for second opinions on initial diagnoses).
2. Demographic characteristics: This part focused on collecting demographic data such as gender, age, and educational background, as well as the patients′ chief complaints.
3. Satisfaction questionnaire: It was developed by a central committee comprising three methodologists with expertise in satisfaction assessment tools. This committee incorporated elements from (1) the Telehealth Satisfaction Scale, (2) the Telemedicine Satisfaction Questionnaire, and (3) the Telemedicine Satisfaction and Usefulness Questionnaire. However, as Ruban lacks online video and voice call functionalities, the existing questionnaires were not suitable for evaluating patient satisfaction with Ruban. Therefore, necessary modifications were made to tailor the questionnaire to this context.
  1. The questionnaire comprised 16 items, scoring by a Likert scale ranging from 1 (indicating *poor satisfaction*) to 10 (indicating *excellent satisfaction*). Although most satisfaction studies use a 5‐ or 7‐point Likert scale, we applied a 10‐point scale to allow finer discrimination of responses. Exploratory factor analysis categorized the questionnaire into four main factors, which we named the subscales as app usability, healthcare provider interaction, advantages and disadvantages of virtual consultation, and future intentions regarding the use of Ruban.

### 2.2. Data Collection

Data were collected by two trained female staff members who conducted interviews via telephone. To ensure privacy, an anonymized connection was established using the Ruban platform′s secure system, so that neither party could identify the other. Interviewers followed a standardized script and maintained a neutral, nondirective tone to minimize response bias. Each patient was contacted once for study participation. Following a brief explanation of the study, consent for participation was sought. The questionnaire was administered verbally only to those who consented. Participants were assured that their responses would remain confidential and would not affect their medical care. To enhance clarity and transparency, we present an example of the individual survey item scores alongside the composite scores in Table S1. Although the primary findings of this study are based on composite measures, displaying the item‐level responses allows readers and reviewers to better understand the domains assessed, the distribution of responses, and how the composite scores were derived.

### 2.3. Statistical Analysis

Descriptive statistics were presented as raw numbers, percentages, mean, and standard deviation or median and interquartile range, whichever was appropriate. Univariate analysis was done using the chi‐square test. We conducted an exploratory factor analysis on the satisfaction questionnaire′s items. Its internal consistency was assessed by Cronbach′s alpha coefficient.

## 3. Results

Among 583 eligible cases, 367 (62.9%) consented to participate, 28 (4.8%) declined, 63 (10.8%) did not recall the consultation clearly, and 125 (21.5%) were unreachable or did not respond to the call.

In total, we interviewed 367 patients, the majority of whom were female and had a higher education (76%). Those without a history of teleconsultation nearly doubled in number (62.4%) compared with those with previous experience (37.6%). The vast majority of patients lived with other members of their family (83.1%) and predominantly used the Ruban app either to seek a second opinion (61.6%) or to follow up on their conditions (51.8%). Their primary complaints included breast pain (39%), erythema (19.6%), ulcer (17.7%), mass (the most common complaint, at 50.7%), discharge (0.8%), nipple retraction (3%), and asymptomatic cases (15.5%) (Table 1).

**Table 1:** Characteristics of the studied variables.

**Variable**	**Overall (** **N** = 367**)**
Age mean (SD)	36.77 (9.85)
Age median (IQR)	35.00 (14.00)
Age *n* (%)	
< 35	173 (47.1)
≥ 35	194 (52.9)
Sex *n* (%)	
Female	363 (98.9)
Male	4 (1.1)
Education *n* (%)	
≤ 12 years	88 (24.0)
> 12 years	279 (76.0)
Contact person is the patient *n* (%)	
Yes	270 (73.6)
No	97 (26.4)
History of teleconsultation *n* (%)	
Yes	138 (37.6)
No	229 (62.4)
Living status *n* (%)	
Alone	42 (11.4)
With others	305 (83.1)
Disease *n* (%)	
Granulomatous mastitis	94 (25.6)
Fibrocystic change	81 (22.1)
Adenoma	42 (11.4)
Breast cancer	79 (21.5)
Infection	9 (2.5)
Others	25 (6.8)
Unknown	37 (10.1)
Symptoms *n* (%)	
Pain	143 (39.0)
Erythema	72 (19.6)
Ulcer	65 (17.7)
Mass	186 (50.7)
Discharge	3 (0.8)
Nipple retraction	11 (3.0)
Asymptomatic	57 (15.5)
Reason for using Ruban *n* (%)	
New complaint	51 (13.9)
Treatment	28 (7.6)
Update information	98 (26.7)
Follow‐up	190 (51.8)
Second opinion	226 (61.6)
Only update information	34 (9.3)
Not only update information	333 (90.7)

The mean satisfaction score among all patients was 91.6 (SD = 8), with the lowest reported score being 39.3 and the highest score reaching 100. The median satisfaction score was 92.3 (IQR = 10.3). The Cronbach′s alpha for the overall satisfaction score was 0.77, for the first component score was 0.78, for the second component score was 0.48, and for the third component score was 0.80.

Our univariate analysis revealed that age, sex, and education levels were not significantly associated with satisfaction scores. However, a history of teleconsultation was linked to lower satisfaction scores. Patients who were asymptomatic or did not have erythema reported higher satisfaction scores. Similarly, the absence of a mass and the request for treatment were associated with increased satisfaction scores. In contrast, seeking a second opinion correlated with lower satisfaction scores (Table 2).

**Table 2:** Determinants of total calculated score in all patients in univariable analysis.

**Variable**	**Satisfaction score**	**Mean**	**SD**	**Median**	**IQR**
Age					
< 35	92.14	7.21	92.31	8.76	
≥ 35	91.01	8.80	91.45	11.11	
Sex					
Female	91.57	8.03	92.31	9.41	
Male	95.09	5.69	95.30	10.04	
Education					
≤ 12 years	91.96	7.09	92.31	10.04	
> 12 years	91.49	8.28	92.31	9.41	
If the contact person was the patient					
Yes	91.54	8.56	92.31	10.26	
No	91.80	6.27	91.45	9.41	
History of teleconsultation∗∗					
No	93.78	9.30	96.58	8.76	
Yes	90.30	6.81	90.60	7.69	
Living status					
Alone	93.00	4.66	92.31	7.26	
With others	91.42	8.33	92.31	10.26	
Disease					
Breast cancer	91.59	5.44	91.45	8.55	
Other diseases	92.29	7.20	92.31	9.40	
Any symptoms∗∗					
No	94.44	10.12	98.29	6.41	
Yes	91.08	7.46	91.45	8.55	
Pain					
No	91.42	8.81	92.31	10.26	
Yes	91.90	6.58	92.31	7.69	
Erythema∗∗					
No	92.15	7.76	92.31	9.4	
Yes	89.38	8.66	90.60	7.48	
Ulcer					
No	91.77	8.26	92.31	10.26	
Yes	90.82	6.70	90.60	6.84	
Mass∗∗					
No	92.83	7.92	94.87	8.98	
Yes	90.41	7.93	90.60	8.55	
Reason for using Ruban∗					
Only update information	92.61	11.72	95.73	10.26	
Not only update information	91.50	7.54	92.31	9.41	
New complaint					
No	91.64	7.89	92.31	10.05	
Yes	91.37	8.76	92.31	10.26	
Treatment∗					
No	91.40	8.01	91.45	9.41	
Yes	94.08	7.68	96.15	7.90	
Update information					
No	91.56	7.88	92.31	10.26	
Yes	91.72	8.39	91.88	9.62	
Follow‐up					
No	91.27	9.74	93.16	10.26	
Yes	91.91	5.97	91.45	8.55	
Second opinion∗∗					
No	92.95	8.79	95.73	10.26	
Yes	90.76	7.37	91.45	7.91	

Significance checked with Mann–Whitney test. ∗Demonstrates *p* value < 0.05; ∗∗demonstrates *p* value < 0.01.

In this study, we explored the correlation between user satisfaction scores across three subscales of app usability (App Score), healthcare provider interaction (Physician Score), and the perceived advantages and disadvantages of virtual consultation (Pros and Cons Score). Our analysis further included assessments of Total Calculated Score, Willingness to Use Again, Willingness to Suggest to Others, and Overall Satisfaction. Statistical analysis revealed that both the App Score and Physician Score received high mean evaluations of 92.0 and 94.1, respectively, suggesting strong satisfaction with the app′s usability and the quality of healthcare provider interactions. The Pros and Cons Score, while slightly lower, still indicated a positive reception with a mean of 89.3. The Total Calculated Score, aggregating these components, reflected a high level of satisfaction with a mean of 91.6. The willingness of participants to reuse the app (mean = 92.2) and recommend it to others (mean = 92.9) further underscores the app′s perceived value. Overall Satisfaction, with a mean score of 89.7, also aligns closely with these findings (Table 3).

**Table 3:** Satisfaction scores by subscale.

**Subscale**	**Mean**	**SD**	**Min**	**Max**	**Median**	**IQR**
App Score	92.0	9.3	27.8	100	94.4	13.9
Physician Score	94.1	7.6	33.3	100	94.4	8.3
Pros and Cons Score	89.3	11.1	37.8	100	91.1	13.3
Total Calculated Score	91.6	8.0	39.3	100	92.3	10.3
Willingness to Use Again	92.2	9.9	44.4	100	88.9	11.1
Willingness to Suggest to Others	92.9	9.5	33.3	100	100	11.1
Overall Satisfaction	89.7	11.4	0	100	88.9	11.1

## 4. Discussion

Telehealth has emerged as a transformative solution, especially in the wake of the COVID‐19 pandemic, offering vast potential to bridge healthcare disparities. With lockdowns and social distancing measures in place, traditional follow‐ups for chronic diseases faced significant disruptions, leading healthcare providers worldwide to pivot towards teleconsultation services. This shift not only ensured continuity of care for patients unable to visit healthcare facilities in person but also presented a unique opportunity to rigorously evaluate the effectiveness of virtual consultations. This research, along with others, could demonstrate the effectiveness of this new communication method and high patient satisfaction, making it useful even beyond outbreaks and pandemics.

We assessed the satisfaction of the patients regarding various parts of the consultation, the physicians, the application, and their overall satisfaction with the virtual consultation. The data collectively indicate a strong positive correlation between app usability, healthcare provider interaction, and the overall advantages of virtual consultation, with these factors contributing significantly to user satisfaction and the likelihood of continued use and recommendation of the app. Based on the findings, providing virtual consultations to patients requires a user‐friendly app and physicians who are interested in offering this type of service.

Moreover, there was no significant difference between age and the satisfaction score. In a similar study assessing the satisfaction of 396 patients with teleconsultation during the COVID‐19 pandemic, Jannati et al. also found no significant relationship between age and overall satisfaction [26]. This contrasts with the findings of a 2021 study by Bizot et al., which followed 1299 breast cancer patients using telephone and video consultations to monitor their treatment outcomes. They reported that age played a crucial role in patient satisfaction, as those under the age of 40 showed a higher satisfaction score with the teleconsultation [11]. This discrepancy could be attributed to the increased adaptability to technology during the COVID‐19 pandemic, suggesting that our study′s context may have influenced its outcomes. It is noteworthy to consider whether our methodology or the timing of our study, relative to ongoing global events, played a role in these divergent findings. Furthermore, more than a quarter of our patients completed the consultation with the help of a relative. It is likely that this group sought assistance from someone more experienced with telecommunication tools, such as websites and apps. This can justify the overall satisfaction in our study that was not influenced by the patients′ age or education level.

Furthermore, our results indicated that patients receiving teleconsultation for the first time reported higher satisfaction scores compared with those with prior teleconsultation experience (93.78 vs. 90.30). The higher satisfaction scores among the first‐time teleconsultation patients in our study could be attributed to several factors: First‐time users might have had lower expectations or no baseline experience for comparison, making them more likely to be impressed with the convenience and efficiency of the service. The novelty of the experience could also contribute to a more positive perception. On the other hand, the patients with prior teleconsultation experiences might have higher expectations based on their past interactions, leading them to be more critical of the services received. Furthermore, they may compare the current service with the previous ones, focusing on any perceived deficiencies or areas where the service did not meet their expectations based on their past experiences. This comparison could influence their satisfaction scores negatively, even if the service quality were objectively high. Other studies have also confirmed that the characteristics of the patient population, such as their previous experiences with teleconsultation, play a role in determining satisfaction [27].

The results showed that the absence of symptoms was associated with higher satisfaction scores. More specifically, the absence of erythema and mass was associated with higher satisfaction scores. The presence of symptoms and their potential impact on the quality of life of the patients is also supported by Renzi et al. in a study on 396 patients analyzing factors associated with patient satisfaction with care among dermatological outpatients [28]. The findings of our study and the study done by Renzi et al. can be potentially justified by the fact that asymptomatic patients are likely to perceive their health status more positively, leading to higher satisfaction with healthcare services because their quality of life is not adversely affected by physical discomfort or mental stress related to symptoms. Furthermore, patients experiencing symptoms are generally more anxious and expect quicker responses. Because our application was offline and physicians had a 24‐h response period, this delay contributed to relative dissatisfaction among symptomatic, more anxious patients regarding the care they received.

Our study found that patients who requested treatment had higher satisfaction scores compared with individuals who did not request treatment. This indicates a positive correlation between treatment requests and satisfaction with healthcare services. In fact, the observed correlation between patients requesting treatment during teleconsultation and higher satisfaction scores can be justified by considering the interplay of trust and the proactive engagement of patients in their care. Other research findings indicate that the prescription of new medication, a proactive form of treatment, was more frequent among patients who exhibited higher levels of trust in their healthcare providers [29]. This trust is a crucial factor in patient satisfaction, suggesting that when patients feel confident in their providers′ ability to manage their health effectively, they are more likely to be satisfied with the care they receive. The act of requesting treatment might signal a higher engagement and trust in the telehealth process, aligning with findings that show a direct relationship between trust and the frequency of proactive treatments like new prescriptions [29]. Patients who are more engaged and trusting are likely to have more positive expectations and perceive their care as more tailored and responsive to their needs, leading to higher satisfaction scores. Conversely, patients with a low level of trust, who might be less inclined to seek or request specific treatments, were found to be less satisfied with their care. This suggests that trust not only facilitates a more active patient role but also enhances satisfaction by fostering a sense of collaboration and personalized care in the telehealth setting. Also, requesting a second opinion was associated with a lower satisfaction score (90.75 vs. 92.95). This finding can partially be explained by the fact that patients often pursue a second opinion in medical matters when their initial diagnosis lacks clarity or when the treatment options available are complex, undesirable, or carry potential risks, which can independently lead to decreased satisfaction ratings [30]. Lower satisfaction among patients seeking a second opinion may be due to the nature of their disease, the complexity of the treatment, and miscommunication with their initial physician. Moreover, in cases where the second opinion differs from that of the treating physician, it can heighten uncertainty in the patient′s decision‐making process.

Overall, our study found high levels of patient satisfaction with teleconsultation services, suggesting that virtual visits are both feasible and well‐accepted in the context of breast healthcare. These findings highlight the potential of telemedicine to expand access to specialist care and to provide a satisfactory alternative to in‐person visits, especially in resource‐limited settings. Although satisfaction is a critical component of care delivery, future studies should evaluate clinical outcomes to determine whether this modality can improve health results. These results are also supported by other research findings. Taxonera et al. conducted a study on 212 IBD patients in 2021 whose face‐to‐face care scheduled visits were converted to remote telephone visits, and they also had 162 urgent remote telephone visits. They evaluated 171 patients (including both scheduled [123] and urgent visits [48]) using a four‐item telephone survey. The patients showed a great degree of satisfaction with the telephone consultation with a 94% satisfaction rate for scheduled visits and 93% for urgent consultations [12]. Also, Li et al. conducted a study in 2020 to evaluate the effectiveness and patient satisfaction of telemedicine virtual communications in vascular patients during the COVID‐19 pandemic for 114 patients in which all patients were satisfied or highly satisfied with the care they received and would use the teleconsultation method again [31].

In this study, we used offline messaging as a consultation method for patients with nonurgent chronic breast complaints. The use of offline messaging offers both advantages and limitations. The advantages of using offline messaging for teleconsultation include increased flexibility for patients and healthcare providers, as individuals can send and receive messages at their convenience without needing to align schedules for a live session. This method can reduce waiting times and make healthcare more accessible, especially for those with busy schedules or living in remote areas. It also allows for thoughtful communication, as both parties have more time to consider their responses.

However, limitations include potential delays in emergency situations, as real‐time interaction is not available. Due to the lack of verbal cues and immediate feedback, there might be misunderstandings or miscommunications. Additionally, nuanced or complex medical issues may not be as effectively communicated or understood through text alone. Further, another limitation of our study is the use of orally administered questionnaires, which may introduce social desirability bias. Although we utilized anonymized phone connections and trained female staff to reduce pressure, participants may still have been influenced by the presence of an interviewer. This is particularly relevant in cultural contexts where gender dynamics may affect how freely female respondents express criticism. Future studies may benefit from using self‐administered electronic surveys to mitigate this potential bias.

Another potential limitation of this study is the selection bias towards patients who were already predisposed to accept and engage in teleconsultation. This means the sample may not fully represent the broader patient population, particularly those less comfortable or unfamiliar with telemedicine. However, it is noteworthy that the study still provides valuable insights as the high satisfaction scores indicate that the service is meeting or exceeding the expectations of those who chose to participate, including a significant portion who were trying teleconsultation for the first time. This suggests a level of effectiveness and acceptance, particularly in the context of increased technology use driven by COVID‐19. To address this limitation in future studies, efforts should be made to include a more diverse group of patients, including those less inclined towards telemedicine, to gain a more comprehensive understanding of patient satisfaction. Additionally, continued promotion and education about teleconsultation can help reduce hesitancy and encourage broader participation, thereby providing a more accurate reflection of patient satisfaction across the healthcare spectrum.

In conclusion, telehealth has emerged as a powerful tool in bridging the gap in healthcare disparities, particularly in underserved areas. By leveraging technology to deliver medical care and consultation remotely, telehealth offers access to quality healthcare services for populations that might otherwise be excluded due to geographic, economic, or systemic barriers. The fact that participants in our study used this platform from various regions across the country, including both urban and rural areas, and received opinions from top specialists working in major referral centers in the capital serves as evidence for this claim though this needs more comprehensive research in the future to be applied in different services.

Our study′s findings indicate that the patients are highly satisfied with their telehealth experiences, suggesting that virtual visits are feasible in meeting the patients′ healthcare needs. This high level of satisfaction among patients underscores the potential of telehealth to make significant inroads in addressing healthcare inequities. Looking ahead, our research can be expanded to more precisely examine the effectiveness of virtual consultations for specific medical conditions. Such a focused analysis could identify conditions for which virtual visits can completely obviate the need for in‐person consultations. By pinpointing these conditions, healthcare providers can tailor their telehealth services more effectively, further enhancing access to care and potentially reducing healthcare costs. This direction not only promises to refine the application of telehealth but also to deepen our understanding of its role in a more equitable healthcare system.

## Ethics Statement

The study was conducted in accordance with the ethical standards of the institutional research committee and with the 1964 Helsinki Declaration and its later amendments.

## Disclosure

All authors read and approved the final manuscript.

## Conflicts of Interest

The authors declare no conflicts of interest.

## Author Contributions

Y.S.J. contributed to data analysis, interpretation, manuscript drafting, and preparing the final version of the manuscript. S.A. contributed to the study design, data collection, and manuscript drafting. N.R. and F.K. contributed to data gathering and interpretation. S.H. supervised data analysis and contributed to the interpretation and finalizing of the manuscript. A.K. provided the first idea of the project and supervised the whole project from design to final manuscript.

## Funding

No funding was received for this manuscript.

## General Statement

This manuscript adheres to the Strengthening the Reporting of Observational Studies in Epidemiology (STROBE) guidelines for reporting observational studies.

## Supporting information


**Supporting Information** Additional supporting information can be found online in the Supporting Information section. Table S1: Example individual survey item responses (P1–P5). Patient‐level scores (Scale 1–10; higher scores indicate a more favorable response) for convenience, security, enough time with clinician, properly convey concerns, clinician understood problem, adequate attention, timeliness, explained clearly, saved time, saved money, easy access, physical contact (reverse‐coded), communication with other physicians, willingness to use again, and recommend to others. Values are raw item scores for five example participants (P1–P5). File type: DOCX.

## Data Availability

The data that support the findings of this study are available from the corresponding author upon reasonable request.
