# Supplementary material for: Digital Health for Breast Care: Patient Satisfaction and Reducing Disparities through Telemedicine
Source: Int J Breast Cancer. 2025 Dec 7;2025:1932655. doi: 10.1155/ijbc/1932655 (PMC12752833; doi:10.1155/ijbc/1932655)
Supplement: Supplementary file 1 — Supporting Information Additional supporting information can be found online in the Supporting Information section. Table S1: Example individual survey item responses (P1–P5). Patient‐level scores (Scale 1–10; higher scores indicate a more favorable response) for convenience, security, enough time with clinician, properly convey concerns, clinician understood problem, adequate attention, timeliness, explained clearly, saved time, saved money, easy access, physical contact (reverse‐coded), communication with other physicians, willingness to use again, and recommend to others. Values are raw item scores for five example participants (P1–P5). File type: DOCX. [file IJBC-2025-1932655-s001.docx]

**Supplementary Table 1. Example Individual Survey Item Responses and Composite Scores**

| Survey Item | P1 | P2 | P3 | P4 | P5 |
| --- | --- | --- | --- | --- | --- |
| Convenience | 9 | 7 | 10 | 6 | 8 |
| Secure | 8 | 6 | 9 | 5 | 7 |
| Enough_time | 9 | 7 | 10 | 6 | 8 |
| Properly_convey | 8 | 6 | 9 | 5 | 7 |
| Understand_problem | 9 | 7 | 10 | 6 | 8 |
| Adequate_attention | 10 | 8 | 9 | 7 | 8 |
| Timeliness | 9 | 6 | 10 | 5 | 8 |
| Explain_clearly | 9 | 7 | 10 | 6 | 8 |
| Saving_time | 8 | 7 | 9 | 6 | 7 |
| Saving_money | 7 | 5 | 8 | 4 | 6 |
| Easy_access | 9 | 8 | 10 | 6 | 8 |
| Physical_contact | 6 | 5 | 7 | 4 | 6 |
| Communication_other_physicians | 8 | 6 | 9 | 5 | 7 |
| Willingness_again | 9 | 7 | 10 | 6 | 8 |
| Suggest_others | 9 | 6 | 10 | 5 | 8 |
| Overall_satisfied | 9 | 7 | 10 | 6 | 8 |
| Composite Score (mean) | 8.5 | 6.7 | 9.4 | 5.6 | 7.5 |
